# Omitted Study Rationale and Acknowledgment of Race as a Social Construct

**DOI:** 10.1001/jamanetworkopen.2023.19505

**Published:** 2023-05-12

In the Research Letter “Effectiveness and Safety of Dapagliflozin for Black vs White Patients With Chronic Kidney Disease in North and South America: A Secondary Analysis of a Randomized Clinical Trial,”^1^ published April 27, 2023, there were several important omissions and errors that require correction. The published article did not include the study rationale for this analysis, which was omitted during revision. The correct rationale is “Relative to White patients, Black patients in North and South America often experience obstacles in accessing high-quality health care, including structural racism, and, in general, experience a distinct array of comorbid conditions and social determinants of health that can affect longevity, disease trajectory, health-related quality of life, and response to therapeutic interventions.” Additionally, the study hypothesis was “because of previously reported differences in experiences regarding comorbidities, barriers to care, and social determinants of health, there could be differences in reported findings of effectiveness and safety of SGLT2 inhibitors among Black and White patients with CKD.” In addition, the article did not acknowledge that race is a social construct, and the title included “Black vs White Patients,” instead of “Black and White Patients.” The Research Letter has been corrected, and the editors have published an Editorial to explain what happened and to apologize to readers.^2^
